# St. John’s Wort Extract Ze 117 and Escitalopram Alter Plasma and Hippocampal Lipidome in a Rat Model of Chronic-Stress-Induced Depression

**DOI:** 10.3390/ijms252312667

**Published:** 2024-11-26

**Authors:** Hendrik Bussmann, Swen Bremer, Anne Marie Hernier, Jürgen Drewe, Hanns Häberlein, Sebastian Franken, Virginie Freytag, Georg Boonen, Veronika Butterweck

**Affiliations:** 1 Medical Department, Max Zeller Soehne AG, Seeblickstrasse 4, 8590 Romanshorn, Switzerland; hendrik.bussmann@zellerag.ch (H.B.); juergen.drewe@zellerag.ch (J.D.); georg.boonen@zellerag.ch (G.B.); 2 Institute of Biochemistry and Molecular Biology, Medical Faculty, University of Bonn, Nussallee 11, 53115 Bonn, Germany; sbremer2192@gmail.com (S.B.); haeberlein@uni-bonn.de (H.H.); sebastian.franken@h-brs.de (S.F.); 3 Porsolt SAS, Z.A. de Glatigné, 53940 Le Genest-Saint-Isle, France; amhernier@porsolt.com; 4 Division of Molecular Neuroscience, Department of Biomedicine, University of Basel, Birmannsgasse 8, 4055 Basel, Switzerland; virginie.freytag@unibas.ch; 5 GeneGuide AG, Birmannsgasse 8, 4055 Basel, Switzerland

**Keywords:** St. John’s wort, depression, lipidomics, chronic stress, Ze 117, biomarker

## Abstract

Chronic stress is a key factor in the development of depression. It leads to hyperactivation of the hypothalamic–pituitary–adrenal (HPA) axis, which in turn increases the formation of glucocorticoids (GCs). Chronically elevated GC levels disrupt neuroplasticity and affect brain lipid metabolism, which may, ultimately, contribute to the development of depression. This study aimed to investigate the effects of the antidepressants St. John’s Wort extract and escitalopram on lipid metabolism in vivo. Therefore, repeated corticosterone injections were used to induce depression-like behavior in rats. Male Sprague–Dawley rats were stressed with corticosterone injections (40 mg/kg, s.c.) over 22 consecutive days and were concomitantly treated with varying doses of the St. John’s wort extract Ze 117 (30, 90 or 180 mg/kg, p.o.) or escitalopram (10 mg/kg, p.o.) and behavioral changes were evaluated using a modified forced swim test. The results indicate that repeated corticosterone injections significantly decreased the latency to first immobility. Furthermore, co-treatment of corticosterone with Ze 117 increased latency to first immobility significantly compared to rats treated with corticosterone alone. To further investigate the biochemical effects of corticosterone-induced stress, as well as the possible counter-regulation by antidepressants, the lipidomes of the plasma and hippocampus samples were analyzed by shotgun mass spectrometry. Corticosterone-induced stress significantly altered key lipid metabolites in the plasma but not in the hippocampal samples. In the hippocampus, however, specific glycerophospholipids such as lysophosphatidylethanolamines (LPEs) increased with escitalopram treatment and with Ze 117, both showing significant correlations with behavioral parameters. In summary, our study shows significant behavioral- and lipidome-altering processes with Ze 117 and escitalopram in rat plasma and hippocampal samples, thereby providing new targets and biomarker ideas for clinical diagnosis and antidepressant intervention.

## 1. Introduction

It is well established that chronic stress has been linked to neuropsychiatric illnesses such as depression [1,2,3]. Chronic stress activates the hypothalamic–pituitary-adrenal axis (HPA) resulting in elevated cortisol levels in patients [4]. Elevated cortisol levels can cause depressive symptoms, which has been demonstrated in patients with Cushing disease and in patients receiving glucocorticoid therapy (for a review, see [5,6]). To understand better the pathophysiology of depression, several animal models have been established which allow for describing the behavioral changes in and molecular effects on brain structures at the same time (for a review, see [7]). One experimental model used to study the relationship between stress, depression and biochemical changes in the brain is chronic exposure of rodents to exogenous corticosterone (equivalent to cortisol in humans) [8]. It has previously been shown that chronic exposure to corticosterone not only induces changes in animal behavior but also leads to molecular and functional alterations in specific brain regions, particularly in the hippocampus [9,10,11]. In addition, several studies provide evidence that chronic stress and the resulting elevated levels of glucocorticoids lead to changes in brain lipid metabolism [12,13,14,15]. The role of lipids in the pathophysiology of stress-related disorders such as depression has been increasingly recognized in recent years [16,17,18]. Lipids within the brain play pivotal roles in providing a physical barrier and dictating the positioning and role of proteins within the cellular membrane. This ultimately governs the synaptic efficiency in neurons [18]. The major lipid structures are glycerophospholipids (phosphatidylcholine (PC), phosphatidylethanolamine (PE), phosphatidylserine (PS), phosphatidylinositol (PI) and phosphatidic acid (PA)); sphingolipids (sphingomyelin (SM) and glycosphingolipids); and sterols (cholesterol), whose relative proportions vary in the brain [19]. Upon signaling, hydrolysis of glycerolipids and sphingolipids is induced. This results in various messenger lipids such as lysophoshatidylcholine (LPC), lysophosphatidylethanolamine (LPE), lysophosphatidylserine (LPS), lysophoshatidic acid (LPA), diacylglycerol (DAG), sphingosylphosphorylcholine (SPC), sphingosine (Sph), sphingosine-1-phosphate (S1P), Cer-1-phosphate (C1P) and ceramides (CER) [17,18,19]. The role of these signaling lipids is important as they either regulate protein recruitment or affect biophysical membrane properties [20,21]. A connection exists between atypical lipid metabolism and depression, possibly through dysregulated neurotransmission and/or neurogenesis [21,22].

In our recent research, we demonstrated that the antidepressant effects of St. John’s wort extract Ze 117 can in part be explained by changes in lipid metabolism [23,24]. In particular, we showed in lipidomics experiments that treatment of stressed peripheral blood mononuclear cells (PBMCs) with Ze 117 increased membrane rigidity—possibly by reducing the average number of double bonds and shortening the chain lengths of fatty acids [23].

Since these previous data were generated using established in vitro stress models, it was of interest in the present study to investigate whether the changes in lipid metabolism can be transferred to an in vivo situation in which a chronic stress model in rats is applied. In the current study, the selective serotonin reuptake inhibitor (SSRI) escitalopram (10 mg/kg), a widely used antidepressant drug, was used as a comparator. St. John’s wort extract (Ze 117) and escitalopram exhibit distinct mechanisms of action. Escitalopram is a highly selective serotonin reuptake inhibitor. Escitalopram operates through its interaction with the sodium-dependent serotonin transporter protein (SERT) located on the presynaptic neuron. SERT’s primary function is to reabsorb serotonin from the synaptic cleft back into the presynaptic neuron. By inhibiting SERT, escitalopram causes an increase in the levels of serotonin within the synaptic space [25]. St. John’s Wort, in contrast, exhibits a more multifaceted mechanism. It contains components that inhibit the reuptake of serotonin, albeit generally to a lesser extent than SSRIs. Additionally, St. John’s Wort acts on other neurotransmitter systems, such as dopamine and noradrenaline [26]. Furthermore, St. John’s wort modulates the hypothalamic–pituitary–adrenal (HPA) axis response. It exerts anti-inflammatory and neuroprotective properties by reducing pro-inflammatory cytokines and oxidative stress [27]. These aspects of St. John’s wort’s action illustrate its complex pharmacological profile, affecting various biological pathways that contribute to its overall efficacy in treating depression. It was therefore of interest to investigate whether there are any differences or similarities in the effects of escitalopram and Ze 117 on the lipidome. To our knowledge, lipidomics analyses have so far not been performed with escitalopram. This investigation, therefore, also provides insight into the mechanism of action of escitalopram.

The experimental design used in this work was adapted, with modifications, from studies by Johnson et al. [8] and Oliviera et al. [14], using chronic corticosterone injections (40 mg/kg s.c.) as a stressor, the evaluation of depression-like behavior in the forced swim test (FST), and a mass spectrometry-based lipidomic approach to determine changes in lipid metabolism. Using lipidomics, we evaluated the peripheral (plasma) and central (hippocampus) metabolic profiles of corticosterone-stressed rats co-treated with Ze 117 or escitalopram with the aim to obtaining new target and biomarker insights for clinical diagnosis and antidepressant intervention.

## 2. Results

### 2.1. Effects of Ze 117 and Escitalopram Treatment on Corticosterone-Induced Depression-Like Behavior

**Figure 1:**
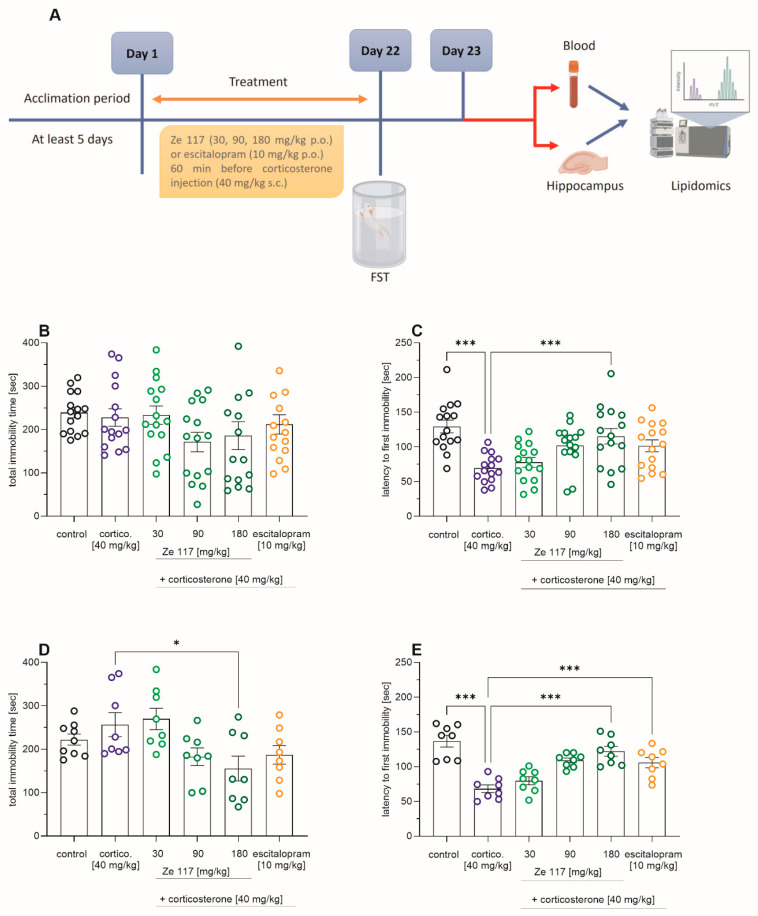
Graphical illustration of the experimental workflow (created with BioRender.com, accessed on 19 January 2024), including treatment schedule and behavioral testing (**A**). Effects of Ze 117 and escitalopram on (**B**) the total immobility time and (**C**) the latency to first immobility of the entire treatment collective (*n* = 15/group). Effects of Ze 117 and escitalopram on (**D**) the total immobility time and (**E**) the latency to first immobility of the selected subgroups (*n* = 8/group). Corticosterone (40 mg/kg) was administered s.c. once daily for 22 days, and Ze 117 (30, 90 and 180 mg/kg, p.o.) and escitalopram (10 mg/kg p.o.) were given to the rats 60 min prior to the corticosterone injection. The values are presented as the mean ± SEM. The levels of significance were determined by one-way ANOVA followed by the Holm–Šídák test for multiple comparisons; * *p* < 0.05 and *** *p* < 0.001.

The experimental setup, as well as the behavioral changes observed during the FST (forced swim test), is shown in Figure 1A. The potential antidepressant effects of Ze 117 and escitalopram were assessed using the FST. Repeated subcutaneous corticosterone injections (40 mg/kg) over 22 days did not affect the total immobility time compared to the vehicle-treated control (Figure 1B) but significantly (*p* < 0.001) reduced the latency to first immobility in male Sprague–Dawley rats (Figure 1C) when the entire treatment collective was analyzed (*n* = 15). In the corticosterone co-treated rats, Ze 117 (180 mg/kg) significantly (*p* < 0.001) increased the latency to first immobility (Figure 1C), from 69.2 ± 5.2 s to 115.4 ± 11.1 s, in the entire population. To perform further lipidomics analyses in a cost-efficient manner, a selection of samples had to be made. Therefore, based on the average mean ± SEM values of the latency of first immobility data (*n* = 15/group), a single sample was drawn from each treatment group to yield a homogenous subgroup with *n* = 8/group. The behavioral parameters of these subgroups were also analyzed (Figure 1D,E).

As illustrated in Figure 1D, in Ze 117 (180 mg/kg) corticosterone-co-treated rats, the total immobility time (155.4 ± 28.5 s) significantly (*p* < 0.05) decreased compared to the corticosterone-only-treated group (256.5 ± 27.7 s). For Ze 117 (90 mg/kg), as well as escitalopram (10 mg/kg), both groups co-treated with corticosterone, there was a trend toward a reduction in total immobility, but this was not significant because of the high level of variability (Figure 1D). Co-treatment of corticosterone [40 mg/kg] with Ze 117 increased the latency to first immobility significantly compared to rats treated with corticosterone alone (Figure 1E). In addition, concomitant treatment of corticosterone and escitalopram significantly increased the latency to first immobility (*p* < 0.001) to 105.7 ± 7.2 s compared to rats treated with corticosterone alone (68.23 ± 5.3 s) (Figure 1E).

### 2.2. Ze 117 and Escitalopram Treatment Induces Changes in the Number of Double Bonds and Chain Lengths

**Figure 2:**
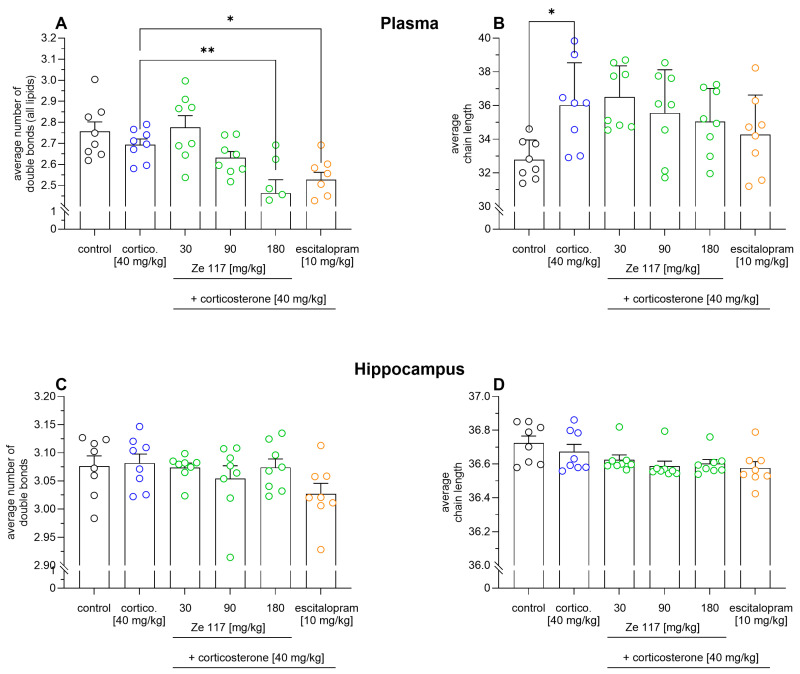
The average number of double bonds and average chain length (±SEM) of all analyzed lipids in the plasma (**A**,**B**) and hippocampus (**C**,**D**) of corticosterone/Ze 117- and corticosterone/escitalopram-exposed rats were compared with the corticosterone condition. Corticosterone (40 mg/kg) was administered s.c. once daily for 22 days, and Ze 117 (30, 90 and 180 mg/kg, p.o.) and escitalopram (10 mg/kg p.o.) were given to the rats 60 min prior to the corticosterone injection. Marked values are significantly different to the corresponding control; * *p* < 0.05 and ** *p* < 0.01, determined by one-way ANOVA, followed by the Holm–Šídák test (*n* = 8).

To find specific markers correlating with behavioral despair in rats, a lipidomic analysis of plasma samples was performed. In the first step, we compared the average number of double bonds and the average chain length within the treatment groups, because in recent lipidomics experiments we showed that the treatment of stressed PBMCs with Ze 117 increased membrane rigidity—possibly by reducing the average number of double bonds and shortening the chain lengths of fatty acids [23]. Since these previous data were generated using an in vitro cell culture system, we wanted to know whether the results could be transferred to an in vivo stress model. However, the average number of double bonds for all plasma lipids did not change after corticosterone administration (Figure 2A) compared to the unstressed control. Interestingly, compared to corticosterone treatment alone, the average number of double bonds in rat plasma lipids decreased significantly, from 2.69 ± 0.08 to 2.46 ± 0.17, when the rats were concomitantly administered corticosterone (40 mg/kg) and Ze 117 (180 mg/kg). A similar effect was observed for concomitant treatment with escitalopram (10 mg/kg) and corticosterone. Furthermore, the average chain length of the rat plasma lipids increased significantly (*p* < 0.05) after corticosterone treatment (40 mg/kg), from 32.78 ± 1.17 to 36.01 ± 2.53 compared to the unstressed control (Figure 2B). The average chain length tended to decrease after corticosterone/Ze 117 treatment to 35.05 ± 1.96 compared to corticosterone alone, but the effect was not significant. This was possibly because of the high variability. A similar trend was observed for corticosterone/escitalopram treatment, which decreased the average chain length to 34.28 ± 2.33. However, no treatment effect was seen on the average number of double bonds and the average chain length in hippocampal probes (Figure 2C,D).

### 2.3. Effects of Ze 117 and Escitalopram Treatment on Key Lipid Metabolites in Plasma and Hippocampus

**Figure 3:**
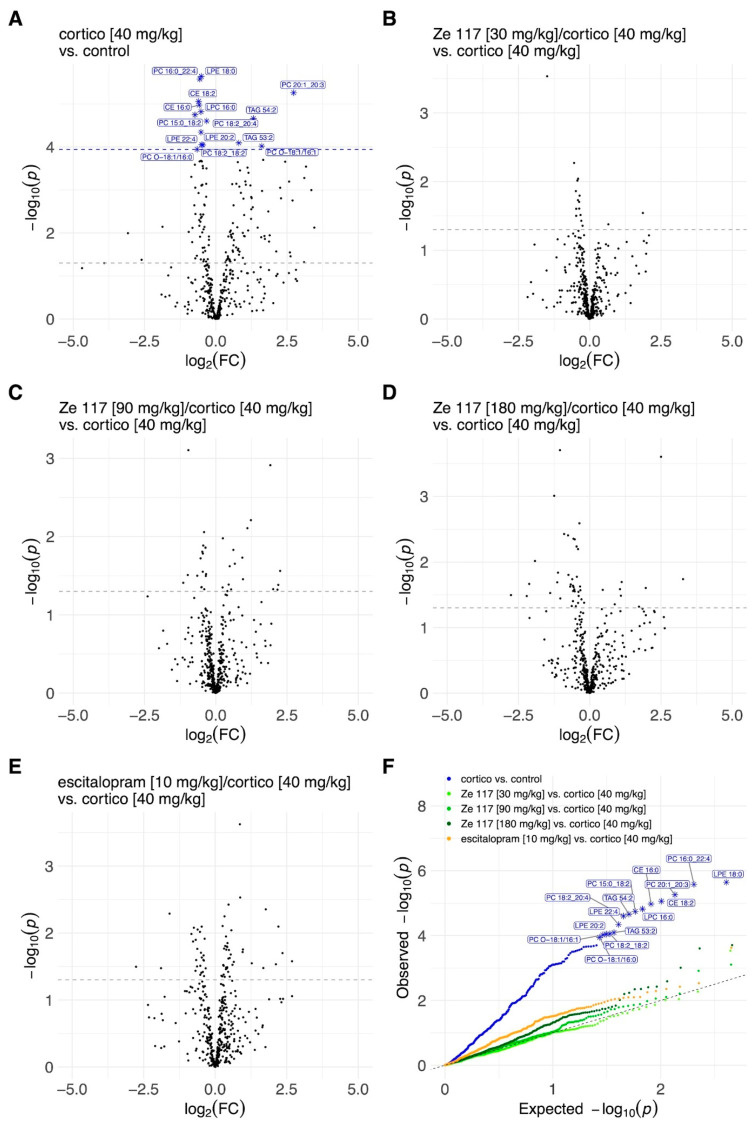
Volcano plots of lipidomics illustrating the different lipid species among treatments in the rat plasma samples: (**A**) corticosterone (40 mg/kg, s.c.) vs. control (distilled water, p.o.); (**B**) Ze 117 (30 mg/kg, p.o.)/corticosterone (40 mg/kg, s.c.) vs. corticosterone (40 mg/kg, s.c.); (**C**) Ze 117 (90 mg/kg, p.o.)/corticosterone (40 mg/kg, s.c.) vs. corticosterone (40 mg/kg, s.c.); (**D**) Ze 117 (180 mg/kg, p.o.)/corticosterone (40 mg/kg, s.c.) vs. corticosterone (40 mg/kg, s.c.); (**E**) escitalopram (10 mg/kg, p.o.)/corticosterone (40 mg/kg, s.c.) vs. corticosterone (40 mg/kg, s.c.). (**A**) Each circle represents one lipid species. The *x*-axis represents log2 (fold change), and the *y*-axis represents −log10 (*p*-value). The line parallel to the *x*-axis displays the *p*-value < 0.05. Significant associations for a given contrast (FDR < 0.05) are highlighted in blue, marked as ‘*’ with the corresponding lipid species label. (**F**) QQ plot of the association *p*-values across different contrasts. Each dot represents the association *p*-value obtained for a single lipid species for a given contrast. Significant associations for a given contrast (FDR < 0.05) are marked with ‘*’ and the corresponding lipid species label.

**Figure 4:**
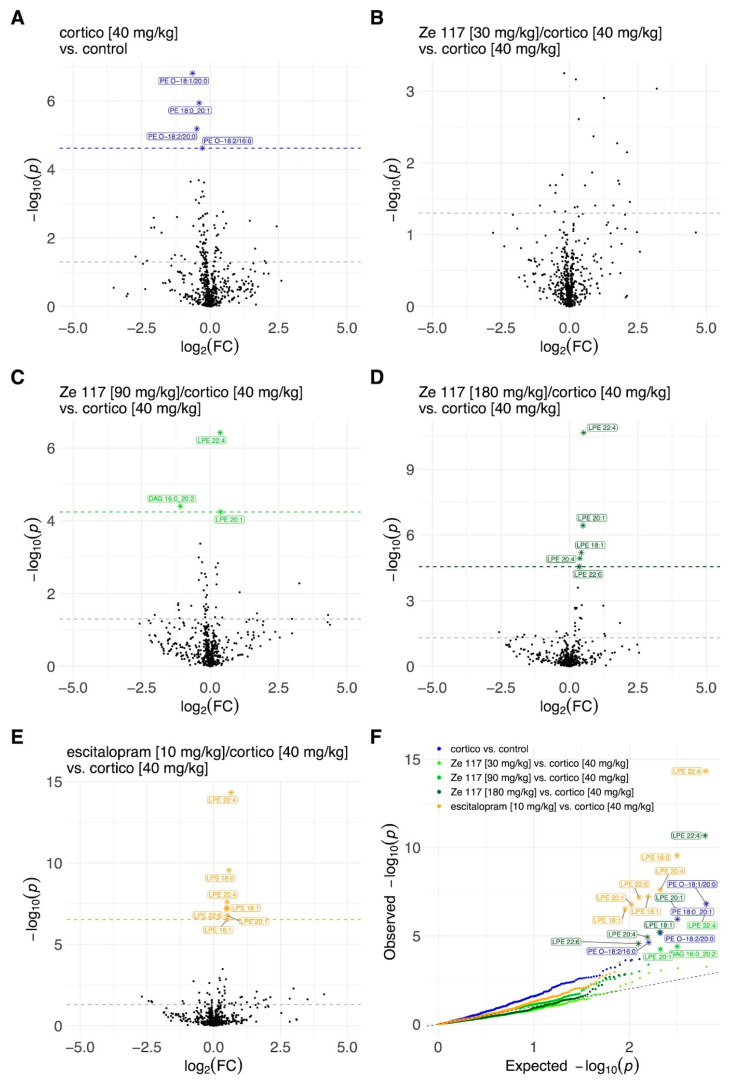
Volcano plots of the lipidomics illustrating the differing lipid species among the treatments in the rat hippocampus samples: (**A**) corticosterone (40 mg/kg, s.c.) vs. control; (**B**) Ze 117 (30 mg/kg, p.o.)/corticosterone (40 mg/kg, s.c.) vs. corticosterone (40 mg/kg, s.c.); (**C**) Ze 117 (90 mg/kg, p.o.)/corticosterone (40 mg/kg, s.c.) vs. corticosterone (40 mg/kg, s.c.); (**D**) Ze 117 (180 mg/kg, p.o.)/corticosterone (40 mg/kg, s.c.) vs. corticosterone (40 mg/kg, s.c.); (**E**) escitalopram (10 mg/kg, p.o.)/corticosterone (40 mg/kg, s.c.) vs. corticosterone (40 mg/kg, s.c.). Each circle represents one lipid species. The *x*-axis represents log2 (fold change), and the *y*-axis represents −log10 (*p*-value). The line parallel to the *x*-axis displays the *p*-value < 0.05. Significant associations for a given contrast (FDR < 0.05) are highlighted in blue, marked with ‘*’ and the corresponding lipid species label. (**F**) QQ plot of the association *p*-values across the different contrasts. Each dot represents the association *p*-value obtained for a single lipid species for a given contrast. Significant associations for a given contrast (FDR < 0.05) are marked with ‘*’ and the corresponding lipid species label. Colors indicated the following treatment groups: blue = corticosterone; green = Ze 117; orange = escitalopram.

To better understand the lipids composition in the plasma and hippocampus, we performed a comprehensive statistical analysis. The alterations in plasma lipid species levels are illustrated using volcano and QQ plots in Figure 3. Subcutaneous corticosterone injections over 22 days (40 mg/kg) significantly upregulated most lipid species compared to plasma samples of unstressed control rats (Figure 3A). No particular pattern of specifically upregulated lipid species was identified. However, when corticosterone treatment was compared to corticosterone co-treatment with Ze 117 or escitalopram, no significant treatment effect was detected (Figure 3B–E). These data were then plausibly confirmed by QQ plots (Figure 3F), illustrating, again, that in the plasma samples, corticosterone-induced stress significantly upregulated key lipid metabolites compared to the unstressed controls but that co-treatment with corticosterone and Ze 117 or escitalopram showed no significant effect when compared to the corticosterone-treated control. In contrast to the plasma samples, the corticosterone treatment alone did not have a strong stress effect at the single-lipid species level in the rat hippocampus samples (Figure 4A). Only selected metabolites such as certain PE-Os were upregulated. However, even if there was no detectable significant corticosterone-induced stress effect in the hippocampus, an effect of Ze 117 treatment was observed in rats co-treated with corticosterone. Ze 117 led to a significant increase in LPE species (Figure 4B–D). However, the higher the dose of Ze 117, the greater the significant increase in lysophosphatidylethanolamine (LPE) species (Figure 4B–D). A similar effect was observed for the fold changes in escitalopram/corticosterone vs. corticosterone (Figure 4E). The QQ plot confirmed the significant treatment-dependent differences in the distribution of LPEs (Figure 4F).

### 2.4. Effect of Ze 117 and Escitalopram Treatment on Specific Lipid Metabolites in Hippocampal Tissue

As mentioned above, we observed an unspecific upregulation of various lipid species in plasma samples after corticosterone treatment compared to the unstressed control but no particular treatment effect when compared to the corticosterone group. Conversely, no stress effect was observed in hippocampal tissue after corticosterone injections but a significant treatment-dependent increase in various LPE species was measured, indicating that Ze 117 and escitalopram act on glycerophospholipid metabolism.

**Figure 5:**
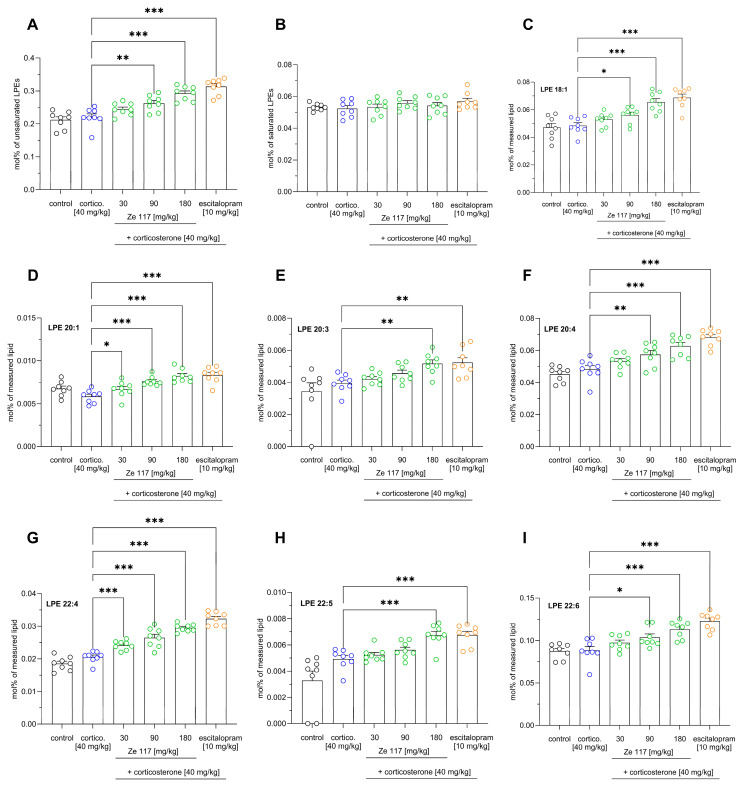
(**A**–**I**) Lysophosphatidylethanolamine (LPE) composition in the hippocampus of corticosterone-stressed rats. Corticosterone (40 mg/kg) was administered s.c. once daily for 22 days, and Ze 117 (30, 90 and 180 mg/kg, p.o.) and escitalopram (10 mg/kg p.o.) were given to the rats 60 min prior to the corticosterone injection. The values are expressed as the mol% ± SEM. The marked values are significantly different from the corresponding control; * *p* < 0.05, ** *p* < 0.01 and *** *p* < 0.001, determined by one-way ANOVA, followed by the Holm–Šídák test (*n* = 8/group).

To further explore this hypothesis, we evaluated the compositions of these specific LPE species in greater detail (Figure 5). Interestingly, the modifications within the LPE class applied only to unsaturated species but not to LPE species with saturated fatty acids (Figure 5A,B). The abundance of unsaturated fatty acids within the LPE class increased significantly (*p* < 0.001), from 0.22 ± 0.01 mol% in the corticosterone-treated (40 mg/kg) rats to 0.29 ± 0.007 mol% in rats co-treated with Ze 117 (180 mg/kg) (Figure 5A). Escitalopram had a similar effect, increasing the abundance of unsaturated LPE species significantly (*p* < 0.001) to 0.31 ± 0.008 mol% (Figure 5A). As shown in Figure 5C–I, several LPE species, including LPE 18:1, LPE 20:1, LPE 20:3, LPE 20:4, LPE 22:4, LPE 22:5, and LPE 22:6, significantly (*p* < 0.01 and *p* < 0.001) increased with the Ze 117 treatment. Interestingly, in the corticosterone- vs. corticosterone/escitalopram-treated rats, the same LPE species significantly (*p* < 0.01 and *p* < 0.001, respectively) increased.

### 2.5. Correlation Analysis Between FST Behavior and Specific LPE Species in the Hippocampus of Corticosterone-Stressed Rats

**Figure 6:**
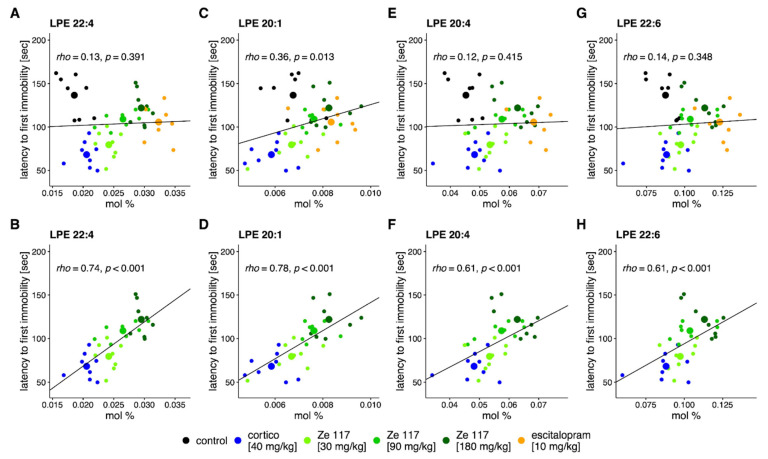
Spearman correlation analysis between the amounts of lipid species (mol%) and the animal’s behavioral measure of latency to first immobility. Top panels (**A**,**C**,**E**,**G**) include samples from all treatment conditions. Bottom panels (**B**,**D**,**F**,**H**) include samples under the corticosterone [40 mg/kg] versus Ze117 (30, 90 or 180 mg/kg) + corticosterone (40 mg/kg) conditions. Each dot represents a single animal. Larger dots represent the mean values within each treatment group, *n* = 8/group. Colors indicate the treatment group (black: control treatment; blue: corticosterone treatment [40 mg/kg]; green: corticosterone + different concentrations of Ze 117 [30–180 mg/kg]; and orange: corticosterone + escitalopram [10 mg/kg]).

As part of the limbic system, the hippocampus plays an important role in mood disorders such as depression. Since we observed an increase in the hippocampus of specific LPE species after Ze 117 or escitalopram treatment in corticosterone-stressed rats, we performed a correlation analysis between the amounts of LPE species and the latency to first immobility. When all treatment conditions including the unstressed control were considered, no correlations between the LPE metabolites and the behavioral parameter FST were found (Figure 6A,C,E,G). However, when the correlation analysis was performed considering the corticosterone and corticosterone/co-treatment conditions, we observed a positive correlation between LPE species and the latency of first immobility (Figure 6B,D,F,H). Notably, a higher content of LPEs in the hippocampus was linked to a higher latency to immobility in the Ze-117-treated rats.

## 3. Discussion

Chronic injections of corticosterone in rodents have frequently been used to induce stress. This leads to behavioral changes reflecting symptoms of depression, such as reduced exploratory behavior, altered sleep patterns, and impaired cognition [8,28,29]. These changes mimic the aspects of depressive-like behaviors observed in humans. In the present study, we used the corticosterone-induced model of stress in rodents in conjunction with antidepressant treatments to explore the effects of the St. John’s wort extract Ze 117 and the selective-serotonin-reuptake inhibitor escitalopram on stress-related behavior. This was performed using the forced swim test (FST) as a readout. In addition, we analyzed whether stress or stress in combination with antidepressant treatment caused alterations in the lipid metabolism of plasma and hippocampal samples. Using a combination of the corticosterone-induced stress model and behavioral analysis, we demonstrate that treatment with Ze 117 increased the latency to first immobility in the FST in the corticosterone-stressed rats. Similar effects were found in rats treated with escitalopram. The FST, established by Porsolt and colleagues in the 1970s [30], is a behavioral test used in preclinical research to assess depressive-like behavior or potential antidepressant effects in rodents. The test is based on the observation that animals subjected to inescapable stress will display a characteristic pattern of behavior. This behavior includes initial escape-oriented behavior followed by a period of immobility thought to represent a form of behavioral despair. The total duration of immobility during a specific period is usually measured and considered an indicator of depressive-like behavior or a response to antidepressant treatments [30].

Interestingly, in our study, the effects of Ze 117 and escitalopram were more pronounced for the latency of first immobility and not for the total immobility time. One possible explanation as to why we did not observe a more pronounced effect on the total immobility time could be that each rat was tested in the FST only once. In the originally described FST by Porsolt, however, rats were placed in the swim tank for 15 min 24 h prior to the main swimming session to increase sensitivity to antidepressant treatment [30]. Our study design was based on the procedures described by Johnson et al. [8], who used the same setup to evaluate behavioral changes based on corticosterone injections rather than swim stress itself. Further, differences in rat strains might explain the different outcomes between our experiments (Sprague–Dawley rats) and the study by Johnson et al. (Long–Evans rats) [8].

The latency to first immobility captures the initial reaction of the subject to the stressful situation [31]. This early response can provide insights into the adaptive or coping mechanisms before a state of immobility sets in [31]. While both parameters are valuable in assessing depressive-like behaviors in the FST, the latency to first immobility appears to be more informative because it captures the early stages of the response, potentially offering deeper insights into the underlying mechanisms [31,32]. The response for the latency to first immobility also seems to be the more sensitive parameter. In our experiments, we received more homogenous results for the total collective (*n* = 15/group), as well as for the collective selected for the later lipidomics analysis (*n* = 8/group), when compared to the total time of immobility.

It is currently not exactly known which ingredients in the Ze 117 extract are responsible for the overall activity observed in the current study. However, since the Ze 117 extract is low in hyperforin at 0.1%, it can be assumed that the observed effects are not due to hyperforin. Furthermore, it has already been shown in earlier studies that hypericin, as well as flavonoids, contributes to the overall pharmacological effects of the extract [33,34,35].

In our latest study, we illustrated how the antidepressant properties of the St. John’s wort extract Ze 117 may be linked to alterations in lipid metabolism [23,24]. Specifically, the lipidomics experiments revealed that administering Ze 117 to stressed PBMCs resulted in increased membrane rigidity. This may have been achieved by decreasing the average count of double bonds and shortening fatty acid chain lengths [23]. Since these previous data were generated in a cell culture model, it was of interest to investigate in a valid animal model of depression whether corticosterone stress has an impact on both parameters in plasma and hippocampus samples.

In our current study, we focused on the hippocampus as a brain region, because it has been shown that it is receptive to changes in lipid modulation induced by stress [15]. In addition, we determined the lipid species in the plasma samples since, because of its easy accessibility, it is the most frequently studied biofluid. Changes in the plasma are expected to mirror changes in other organs [16]. Interestingly, the observed changes in the lipid metabolism in the plasma and hippocampus were not congruent in our study. Compared to the unstressed control, the average number of double bonds of all plasma lipids did not change after corticosterone administration, while the average number of double bonds of the rat plasma lipids decreased significantly after the Ze 117 treatment. Similar effects were seen for concomitant treatment of escitalopram and corticosterone. Conversely, the average chain length of the rat plasma lipids increased significantly after corticosterone treatment compared to the unstressed control. Although a trend toward decreasing the average chain length was detected after the Ze 117 or escitalopram treatment, the effect was not significant. These effects were only observed in plasma samples, while in the hippocampus neither the average number of double bonds nor the average chain length were affected. This suggests a compartmentalized or selective effect of corticosterone on lipid metabolism. As the circulating medium for various molecules including lipids, the plasma reflects systemic changes in the body [16]. However, when the average number of double bonds decreases and the average chain lengths of lipids are reduced in lipidomics research (compared to a control group), this often indicates a change in the composition of fatty acids within the cell membranes or lipid structures [14,36]. These alterations can affect the fluidity, flexibility and stability of the membranes. A decrease in double bonds typically implies a shift toward more saturated fatty acids, potentially leading to increased membrane rigidity. Shorter chain lengths might also contribute to alterations in the membrane properties. These changes can have implications for various cellular functions and physiological processes [14,36]. Membrane fluidity is critical for maintaining proper cell function, including signal transduction, receptor activity and ion channel performance [18]. A reduction in fluidity can positively affect neuronal communication and adaptability, potentially reversing depressive-like behaviors by stabilizing synaptic function and plasticity. Similarly, the decrease in fatty acid chain lengths may influence cell membrane structure and rigidity. The balance between stability and fluidity is essential for membrane function, as both are critical for processes like signal transduction, ion channel activity and receptor function. Alterations in the lipid membrane’s composition and function may disrupt the dynamics of neurotransmitter receptors and signaling complexes. This can impair processes critical for stress adaptation and resilience [21]. Moreover, changes in lipid structure, such as increased saturation and decreased chain length, can influence the cellular response to oxidative stress. Saturated fatty acids are generally less prone to peroxidation than polyunsaturated fatty acids [21]. In light of these findings, we suggest that the structural changes in lipids observed in our study could contribute to improved neuroplasticity and increase resilience to stress, both of which are key features of depressive disorders. While these mechanisms are speculative and warrant further investigation, they provide a plausible framework to connect the biochemical alterations we observed with the behavioral outcomes in our model. In addition, the current observation also supported our previous data that Ze 117 increases membrane rigidity by reducing the average number of double bonds in cortisol-stressed [1 µM] PBMCs [23].

We further investigated whether particular lipid species were altered by corticosterone-induced stress in plasma and hippocampus samples. The volcano plots illustrated significant differences in lipid species between the unstressed control group and the corticosterone/corticosterone plus treatment group. While repeated corticosterone injections led to a significant increase in key lipid metabolites in plasma samples compared to the control group, no treatment effect was seen. In the hippocampus, however, no stress effect on the lipidome after corticosterone injections was observed. The absence of observable changes in the hippocampus, a region of the brain associated with memory and stress regulation, could imply that the effects of corticosterone on lipid metabolism did not directly affect this brain region. Previous studies demonstrated that corticosterone-induced stress leads to heterogenous changes in the lipid metabolism in particular brain regions [14,15]. In the hippocampus, corticosterone-induced stress effects were more pronounced in the ventral than the dorsal hippocampus of rats [15]. However, in the present study, we measured an increase in LPE species after Ze 117 or escitalopram treatment in corticosterone co-treated rats in the hippocampus. The significant increase in lipid metabolites observed in plasma samples, as opposed to the lack of observable changes in the hippocampal lipidome following corticosterone injections, indicates that corticosterone’s effects may manifest differently in these two compartments. This could imply that corticosterone impacts lipid metabolism in a more systemic manner (reflected in plasma) rather than directly within the hippocampal region. It might further be possible that corticosterone has complex, region-specific and compartmentalized effects on lipid metabolism, with systemic changes observable in the plasma and more nuanced or condition-dependent effects in the hippocampus. This compartmentalization could be due to differences in blood–brain barrier permeability, local enzymatic activities, or specific cellular responses within different regions of the brain, including the hippocampus. The observed increase in LPE species in the hippocampus after Ze 117 or escitalopram treatment in corticosterone-treated rats further supports the idea that specific lipid species, such as LPEs, may respond differentially to corticosterone co-treatment. It can be speculated that corticosterone may modulate the hippocampal environment so that treatments such as Ze 117 or escitalopram can induce detectable lipidomic changes, even though no observable stress effect from corticosterone alone in the hippocampus was detected.

Lysophosphatidylethanolamines (LPEs) represent a class of phospholipids linked to depression and related mood disorders [37], though their precise functions are still under investigation. LPEs, derivatives of phosphatidylethanolamine (PE) formed via a phospholipase A-type reaction, constitute minor components within cell membranes [38]. Like other lysophospholipids, LPEs display diverse structural variants based on fatty acid length and unsaturation levels. While LPEs have been detected in human serum at measurable concentrations [39], their exact physiological function remains largely unknown. It has been shown that changes in LPE levels can affect neuronal function and synaptic plasticity, both pivotal in mood disorders like depression [40,41]. Specific LPE species have been associated with neuroprotective mechanisms by potentially shielding neurons from stress-induced damage and contributing to neuroplasticity. In particular, in cultured cortical neurons, palmitoyl-LPE (16:0 LPE) and stearoyl-LPE (18:0 LPE) stimulated neurite outgrowth [40,41].

In our study, a Spearman rank correlation analysis revealed a correlation between the latency to first immobility and the increase in LPE species in the hippocampus. The best correlations were observed for LPE 20:1 and LPE 22:4. While these connections hint at LPEs’ potential involvement in depression, the precise mechanisms and their roles in depressive disorders are still actively under investigation. For example, it has been shown that LPEs can suppress LPS-induced inflammatory responses in microglia cells via inhibition of nicotinamide adenine dinucleotide phosphate oxidase (NOX) [42]. NOX, as a major source of reactive oxygen species (ROS) causes oxidative cell damage leading to progressive neuronal damage [43]. Neuroinflammation is one pathological mechanism leading to neurological diseases such as depression or Alzheimer’s disease [44]. Thus, the Ze 117- and escitalopram-induced shifts in lipid metabolism toward an increase in LPE levels could suppress the inflammatory processes and oxidative stress frequently observed in depression. Consequently, the increase in specific LPE species in the hippocampus might contribute to the therapeutic effects of Ze 117 and escitalopram in alleviating depressive symptoms through prevention of neuroinflammation. Neuroinflammation can impair neurogenesis, particularly in the hippocampus, a brain region essential for emotional regulation and stress resilience [45]. Impaired neurogenesis is associated with a lack of adaptive responses to stress and can result in behavior indicative of despair or helplessness in the FST. Reduced synaptic plasticity also limits an animal’s capacity to respond adaptively to the test environment, thereby contributing to increased immobility. LPE s have shown anti-inflammatory properties that can mitigate the impact of pro-inflammatory cytokines, such as IL-1β, IL-6, and TNF-α, which are involved in neuroinflammation and contribute to neurotransmitter imbalances [42]. It can be speculated that by reducing these cytokine levels, LPEs may help maintain the balance of serotonin, dopamine, and glutamate, supporting more stable mood regulation and potentially reducing immobility in FSTs.

It is worth mentioning in this regard that neurotrophic and anti-inflammatory effects by a St. John’s wort extract were shown in mouse hippocampal HT-22 neurons [46]. Furthermore, St. John’s wort extracts have demonstrated antidepressant-like effects in rodent models of chronic stress and have also shown potential neuroprotective effects involving antioxidant and anti-inflammatory properties (for a review, see [27,47]). In addition, studies suggest that St. John’s wort modulates stress-related hormonal changes by regulating the hypothalamic–pituitary–adrenal (HPA) axis [35,48]. Activation of the HPA-axis during stress can lead to increased inflammation and oxidative stress. These factors can, in turn, influence lipid metabolism, leading to changes in levels of phospholipids, triglycerides, and cholesterol [49]. Changes in lipid metabolism due to stress-induced HPA axis activation might influence brain function, affecting neuronal membranes and cell signaling [17,18,21]. The St. John’s wort extract Ze 117, as well as the standard antidepressant escitalopram, seem to affect not only neuroendocrine but also metabolic aspects in capturing the physiological responses to stress. Further, LPE seems to play an important role in this bidirectional communication. LPEs also have the potential to modulate the hypothalamic–pituitary–adrenal (HPA) axis. Chronic stress and neuroinflammation often lead to an overactive HPA axis, resulting in high levels of stress hormones like corticosterone in animals [50]. LPEs, with their neuroprotective effects, might alleviate this chronic activation of the HPA axis by decreasing neuroinflammatory signals that contribute to heightened stress responses. It can be speculated that increased LPE levels triggered by treatment with Ze 117 or escitalopram may reduce behavioral despair in the FST, improving resilience to stress.

It has also been shown that LPEs contribute to the overall composition and structure of cell membranes [51]. While they represent a smaller fraction compared to other lipids, they are essential for maintaining membrane fluidity and stability [51]. Alterations in LPE levels might affect membrane properties, potentially influencing the functioning of membrane-bound proteins involved in mood regulation and neurotransmission [16,18]. LPEs have also been implicated in processes related to neurotransmission and neuroplasticity, affecting synaptic function and neuronal communication [38,40,41]. The observed increase in LPE species with treatment with Ze 117 and escitalopram in corticosterone-stressed rats might indicate an adaptive response to stress. Cells might produce more LPEs upon treatment with Ze 117 or escitalopram as part of a mechanism to cope with stressors, possibly playing a role in cellular signaling or membrane remodeling to adapt to the challenging conditions. However, understanding the complex relationship between changes in LPE levels and depression necessitates further research to determine their significance and potential as therapeutic targets.

## 4. Material and Methods

### 4.1. Animals

Male Sprague–Dawley rats (Janvier Labs, Le Genest-Saint-Isle, France), 201–240 g in body weight, were used. The rats were delivered 7–10 days before the start of the experiments (first day of treatment: day 1) and housed in groups of 3 or 4 in Makrolon^®^ cages (48 × 37.5 × 21 cm) (Carfil, Out-Turnhout, France) on wood litter with free access to food and water. Environmental enrichment (gnawing and nesting materials) was provided. The animal house was maintained under artificial lighting (12 h) between 7:00 and 19:00 in a controlled, ambient temperature of 22 ± 2 °C and relative humidity between 30 and 70%.

### 4.2. Ethical Statement

All animal experiments were performed at the Porsolt Research Laboratory (Le Genest-Saint-Isle, France) and were conducted in compliance with Animal Health regulations, in particular as follows: (a) Council Directive No. 2010/63/UE of 22 September 2010 on the protection of animals used for scientific purposes and French decree No. 2013-118 of 1 February 2013 on the protection of animals; (b) in accordance with the Porsolt facility accreditation for experimentation (E 53 1031, renewed on 19 April 2016); and (c) in accordance with the recommendations of the Association for Assessment and Accreditation of Laboratory Animal Care (AAALAC) of which the accreditation was granted in June 2012 and renewed in 2018.

### 4.3. Treatments and Treatment Schedule

Rats were randomly assigned to the following treatment groups (*n* = 15 per group): The corticosterone group was given a daily injection subcutaneously, for 22 days, of 40 mg/kg corticosterone (Sigma-Aldrich, St. Louis, MO, USA, ref. no. C2505) suspended in isotonic saline and 2% Tween 80 (Sigma-Aldrich, St. Louis, MO, USA). The corticosterone dose used was based on previous studies [8,28]. The control group was injected daily with vehicle (2% Tween 80 in physiological saline for s.c. application and distilled water for p.o. application) for 22 days only. Subcutaneous (s.c.) injections (corticosterone) were administered at a volume of 5 mL/kg (body weight recorded daily) and oral administration (p.o.) (Ze 117 and escitalopram) were administered at a volume of 10 mL/kg.

A quantified extract from *Hypericum perforatum* low in hyperforin (Ze 117; DER: 4–7:1; extraction solvent ethanol: 57.9% (*V*/*V*); batch number: 191026) with 0.26% hypericin, 0.10% hyperforin, and a total flavonoid content of 10.60% (calculated as rutin) was manufactured according to Ph. Eur. EP 9.3/1874 and provided by Max Zeller Söhne AG, Romanshorn, Switzerland. The extract complied with the well-established use monograph of the Herbal Medicinal Products Committee (HMPC) of the European Medicines Agency (EMA) [52]. A characteristic HPLC chromatogram is provided in the Supplemental Materials (Figure S1). The extract was suspended in distilled water, and 30, 90 or 180 mg/kg of Ze 117 was administered p.o. for 22 consecutive days 60 min before the corticosterone injection. Escitalopram (Seroplex^®^, Lundbeck, Denmark, ref. no. 03400935994110) was dispersed using a mortar and pestle in 0.2% hydroxypropylmethylcellulose (HPMC) in distilled water and was administered (p.o.) at a dose of 10 mg/kg over 22 consecutive days 60 min before the corticosterone. The formulations were continuously stirred with a magnetic stir bar until administration.

### 4.4. Modified Behavioral Despair Test

The method used in this experiment was adapted from Porsolt et al. [30] and in line with the experimental settings described previously by other groups [8,28]. In the classical Porsolt forced swim test (FST), rats are placed in a swim cylinder for 15 min on day 1 to simulate ‘helplessness’. Twenty-four hours later they were placed back in it for 5 to 10 min to measure immobility. In the current experiment, each rat was only tested once, since the aim was to measure exposure to corticosterone injections rather than prior exposure to the swim task itself [8]. The FST was administered to all rats 1 h following their last corticosterone treatment. Briefly, the rats were individually placed in a cylinder (height: 40 cm; diameter: 20 cm) containing water (25 °C) for 10 min. The quantity of water was adjusted in such a way that the animals could touch the bottom of the cylinder with the tip of their hind paws (water depth: ~16.5 cm). The experimental room was illuminated by indirect light. The FST was carried out between 9:00 a.m. and 1:00 p.m. The animal behavior was video recorded over the test period of 10 min. The recorded videos were manually evaluated using AnyMaze behavior tracking software (version 7.13, TakeNote mode, Stoelting Europe, Dublin, Ireland). The observer was blinded to the treatment. Each rat was judged to be immobile when it ceased struggling and remained floating motionless in the water for at least 1 s, only making movements necessary to keep its head above the water’s surface [53]. In addition, for a total cumulative immobility over 10 min, the latency to the first bout of immobility was also recorded starting from the beginning of the test [31,32]. After the swimming session, the rats were dried and returned to their home cage. The water was changed between each test.

### 4.5. Biochemical Analysis

At the end of the experiment (24 h after the last treatment and FST), animals were placed under isoflurane anesthesia and approximately 800 µL of blood was collected by cardiac puncture using a sterile disposable syringe. The blood samples were immediately transferred into pre-labeled tubes containing 40 µL of 25× cOmplete™ Protease Inhibitor Cocktail (Sigma Aldrich, St. Louis, MO, USA, ref.no. 11697498001). The solution of 25× protease inhibitor cocktail was freshly prepared daily by dissolving one tablet in 2 mL of distilled water. After sealing each tube, the blood samples were manually agitated and stored on ice until centrifugation (within 30 min of sampling). The samples were centrifuged at +4 °C and 1500× *g* for 10 min. The entire resultant plasma obtained was immediately transferred to suitably labeled polypropylene tubes (6 aliquots of approximately 55 µL). The tubes were stored upright at approximately −70 °C until further analysis.

Immediately after blood collection, animals were decapitated, their brain quickly removed from the skull and rinsed in physiological saline. The hippocampus was then collected, weighed, placed in pre-labeled vials and frozen in liquid nitrogen until the storage. The vials were stored upright at approximately −70 °C and protected from light until analysis.

### 4.6. Sample Preparation for Lipidomics

The plasma and hippocampus samples were analyzed by the commercial mass spectrometry provider Lipotype GmbH (Dresden, Germany). Th mass-spectrometry-based lipid analysis was performed by Lipotype GmbH, as described by Sampaio et al. [54]. Lipids were extracted using a two-step chloroform/methanol procedure [55]. Samples were spiked with an internal lipid standard mixture. After extraction, the organic phase was transferred to an infusion plate and dried in a speed vacuum concentrator. The 1st-step dry extract was re-suspended in 7.5 mM ammonium acetate in chloroform/methanol/propanol (1:2:4, *V*:*V*:*V*) and the 2nd-step dry extract in a 33% ethanol solution of methylamine in chloroform/methanol (0.003:5:1; *V*:*V*:*V*). All liquid handling steps were performed using Hamilton Robotics STARlet robotic platform with the Anti-Droplet Control feature for organic solvents pipetting.

The mass spectra were acquired on a hybrid quadrupole/Orbitrap mass spectrometer equipped with an automated nano-flow electrospray ion source in both the positive and negative ion modes.

### 4.7. MS Data Acquisition

The samples were analyzed by direct infusion on a Q Exactive Orbitrap mass spectrometer (Thermo Scientific, Reinach, Switzerland) equipped with a TriVersa NanoMate ion source (Advion Biosciences, Ithaka, NY, USA). The samples were analyzed in both the positive and negative ion modes, with a resolution of R_m/z = 200_ = 280,000 for the MS and R_m/z = 200_ = 17,500 for the MSMS experiments, in a single acquisition. The MSMS was triggered by an inclusion list encompassing corresponding MS mass ranges scanned in 1 Da increments [56]. Both MS and MSMS data were combined to monitor CE, DAG and TAG ions as ammonium adducts; PC and PC O-as acetate adducts; and CL, PA, PE, PE O-, PG, PI and PS as deprotonated anions. Only MS was used to monitor LPA, LPE, LPE O-, LPI and LPS as deprotonated anions and Cer, HexCer, SM, LPC and LPC O- as acetate adducts.

### 4.8. Data Analysis and Post-Processing

Data were analyzed with in-house developed lipid identification software based on LipidXplorer (version 1.2.8.1) [57,58]. Data post-processing and normalization were performed using an in-house developed data management system. Only lipid identifications with a signal-to-noise ratio >5 and a signal intensity 5-fold higher than in corresponding blank samples were considered for further data analysis. Prior to normalization and further statistical analysis of lipid species, lipid identifications were filtered according to mass accuracy, occupation threshold, noise and background. Lipid quantification was carried out using internal lipid class standards.

### 4.9. Average Number of Double Bond and Average Chain Length of Fatty Acid Moieties

To calculate the average number of double bonds, the amounts, in pmoles, of individual lipid molecules of a given lipid class were summed to yield the total amount of the lipid class. The amounts of the lipid classes were normalized to the total lipid amount yielding mol% per total lipids.

The average number of double bonds (DB_av_) within a lipid class was calculated by the following equation:

(1) $$DB_{av}=\frac{DB\;mol\%}{\sum_{i=1}^{n}DB_{i}\;mol{\%}_{i}}$$

where DB is the number of double bonds of a lipid species within the lipid class and mol% is the mol% value of the respective lipid species.

The average of the fatty acid chain length (CL_av_) within a lipid class was calculated as follows:

(2) $$CL_{av}=\frac{N_{c}\;mol\%}{\sum_{i=1}^{n}N_{c_{i}}\;mol{\%}_{i}}$$

where N_c_ is the number of carbon atoms of a lipid species within the lipid class and mol% is the mol% value of the respective lipid species.

### 4.10. Statistical Analysis

Statistical analysis of the behavioral data was performed using Prism V.9.4.1 software (GraphPad, Boston, MA, USA). The Kolmogorov–Smirnov test was used to determine the normality of the data distribution. Since the data met the assumption of normality, a one-way ANOVA, followed by the Holm–Šídák test, was performed. All results are presented as the mean ± standard error of the mean (SEM), and *p* < 0.05 was considered a statistically significant difference. Behavioral data from the entire treatment collective (*n* = 15 per group) were evaluated, as well as a subset of each collective (*n* = 8 per group). Each subset was chosen using the latency of first immobility data, excluding top and bottom outliers. These rat samples were also used for further lipidomics analysis.

For the lipidomics results, two independent experiments were performed. Lipidomics profiling yielded a quantification of 953 lipids in the hippocampus rat samples and 696 lipids in the plasma samples. The analyses were conducted on lipid amounts standardized to the total amount of lipid within each sample (mol%) to account for the differences in the total volumes analyzed among samples. Prior to analysis, values below the limit of detection (LOD) were replaced by zero. For each lipid, a single linear model was fitted between the lipid amount (mol%) and treatment (control, corticosterone [40 mg/kg], Ze117 [30 mg/kg] + corticosterone [40 mg/kg, Ze117 90 mg/kg + corticosterone 40 mg/kg, Ze117 180 mg/kg + corticosterone 40 mg/kg, and escitalopram 10 mg/kg + corticosterone 40 mg/kg). The following five contrasts of interest were subsequently calculated using *t*-tests as implemented in the emmeans R (4.4.1) package [59]: (a) corticosterone vs. control; (b) Ze117 [30 mg/kg] + corticosterone [40 mg/kg] vs. corticosterone [40 mg/kg]; (c) Ze117 [90 mg/kg] + corticosterone [40 mg/kg] vs. corticosterone [40 mg/kg]; (d) Ze117 [180 mg/kg] + corticosterone [40 mg/kg] vs. corticosterone [40 mg/kg]; and (e) escitalopram + corticosterone [40 mg/kg] vs. corticosterone [40 mg/kg]. For each contrast, only individual lipids with at least one value > LOD in both conditions were considered for analysis. Adjustment for multiple testing was performed separately for each contrast across all lipids tested using Benjamini–Hochberg FDR correction. Relationships between lipid species amounts (in mol%) and the animal behavioral measure of latency to first immobility were examined using Spearman’s rank correlation test, considering (a) all treatment groups and (b) Ze 117 and Ze 117 corticosterone-co-treated groups only. For statistical analysis of the average number of double bonds, average chain length, as well as the differences in mol% of single LPEs, one way-ANOVA was performed, followed by the Holm–Šídák test using Prism V.9.4.1 software (GraphPad, Boston, USA, USA). All results are presented as the mean ± standard error of the mean (SEM) and *p* < 0.05 was considered as a statistically significant difference.

## 5. Conclusions

Our present study demonstrates that both St. John’s wort extract Ze 117 and escitalopram alleviate depression-like behavior in stressed rats by affecting lipid metabolism in plasma and hippocampus samples. While chronic stress affected glycerophospholipid metabolism in plasma samples, no such changes were noted in the hippocampus. However, both treatments notably increased LPE species in the hippocampus, suggesting they primarily affect central, not peripheral, mechanisms. This alteration in LPE metabolism could influence cell signaling and membrane remodeling. Our results indicate LPEs as potential biomarkers for antidepressant treatment, but further research is needed to fully understand their role and significance in treating depressive disorders.

In conclusion, the findings of this study contribute to a more comprehensive understanding of the mechanism of action of antidepressants and St. John’s wort extract in particular. The reason why some patients respond to therapy and others do not remains unclear. Thus, the identification of biomarkers for the diagnosis and effectiveness of therapies is essential to improve the quality of life of those affected.

## Data Availability

The data that support the findings of this study are available from the corresponding author upon reasonable request.

## Supplementary Materials

The following supporting information can be downloaded at: https://www.mdpi.com/article/10.3390/ijms252312667/s1.
