# Supplementary material for: St. John’s Wort Extract Ze 117 and Escitalopram Alter Plasma and Hippocampal Lipidome in a Rat Model of Chronic-Stress-Induced Depression
Source: Int J Mol Sci. 2024 Nov 26;25(23):12667. doi: 10.3390/ijms252312667 (PMC11641671; doi:10.3390/ijms252312667)
Supplement: Supplementary file 1 [file ijms-25-12667-s001.zip › ijms-3312876-supplementary.pdf]

Lipidomic Insights: St. John's Wort Extract Ze 117 and Escitalopram in a Rat Model of Chronic Stress Induced Depression

Hendrik Bussmann [1], Swen Bremer [2], Anne Marie Hernier [3], Jürgen Drewe [1], Hanns Häberlein [2], Sebastian Franken [2], Virginie Freytag [4,5], Georg Boonen [1] and Veronika Butterweck [1,*]

[1]Medical Department, Max Zeller Soehne AG, Seeblickstrasse 4, 8590 Romanshorn, Switzerland
[2]Institute of Biochemistry and Molecular Biology, Medical Faculty, University of Bonn, Nussallee 11, 53115 Bonn, Germany
[3]Porsolt SAS, Z.A. de Glatigné, 53940 Le Genest-Saint-Isle, France
[4]Division of Molecular Neuroscience, Medical Faculty, Department of Biomedicine, Birmannsgasse 8, 4055 Basel, Switzerland
[5]GeneGuide AG, Birmannsgasse 8, 4055 Basel, Switzerland

***Corresponding author:**

Prof. Dr. Veronika Butterweck
Max Zeller Soehne AG
Medical Department
Seeblickstrasse 4
CH-8596 Romanshorn
Email: veronika.butterweck@zellerag.ch

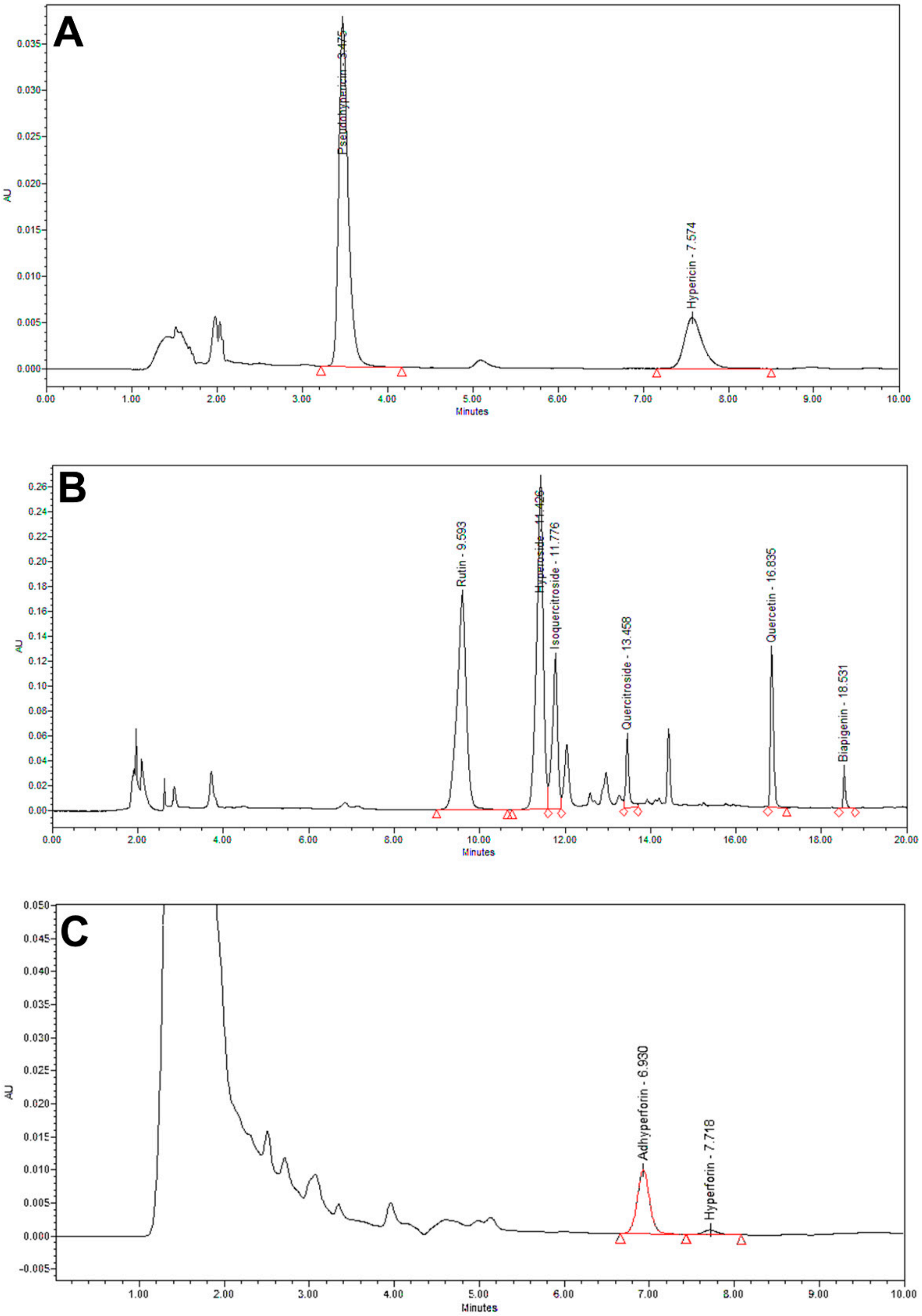

Figure S1: High performance liquid chromatography chromatogram of St. John's wort dry extract Ze 117 (batch 191026). Quantitative determination of total hypericin (pseudohypericin, hypericin), flavonoids (rutoside, hyperoside, isoquercitroside, quercitrin, quercetin and biapigenin) and total hyperforin (adhyperforin, hyperforin), using liquid chromatography with a photo diode array detector (PDA) 590 nm (**A**), 360 nm (**B**) and 274 nm (**C**). A HPLC reversed phase C18 column (e.g. Luna, 150 mm x 4.6 mm, 3 µm) was used.
